# High-throughput LPS profiling as a tool for revealing of bacteriophage infection strategies

**DOI:** 10.1038/s41598-019-39590-8

**Published:** 2019-02-27

**Authors:** Eugene E. Kulikov, Alla K. Golomidova, Nikolai S. Prokhorov, Pavel A. Ivanov, Andrey V. Letarov

**Affiliations:** 1 Winogradsky Institute of Microbiology, Research Center of Biotechnology of the Russian Academy of Sciences, prosp. 60-letiya Oktyabrya, 7 bld. 2, 117312 Moscow, Russian Federation; 2 Moscow Institute of Physics and Technology, Institutskiy per., 9, Dolgoprudny, Moscow Region 141701 Russian Federation; 3 Faculty of Biology, Lomonosov Moscow State University, ul. Leninskie Gory, 1, 119991 Moscow, Russia; 4 Present Address: Department of Biochemistry and Molecular Biology, Sealy Center for Structural Biology and Molecular Biophysics, University of Texas Medical Branch, 301 University Bulevard, Galveston, TX USA

## Abstract

O-antigens of Gram-negative bacteria modulate the interactions of bacterial cells with diverse external factors, including the components of the immune system and bacteriophages. Some phages need to acquire specific adhesins to overcome the O-antigen layer. For other phages, O-antigen is required for phage infection. In this case, interaction of phage receptor binding proteins coupled with enzymatic degradation or modification of the O-antigen is followed by phage infection. Identification of the strategies used by newly isolated phages may be of importance in their consideration for various applications. Here we describe an approach based on screening for host LPS alterations caused by selection by bacteriophages. We describe an optimized LPS profiling procedure that is simple, rapid and suitable for mass screening of mutants. We demonstrate that the phage infection strategies identified using a set of engineered *E. coli* 4 s mutants with impaired or altered LPS synthesis are in good agreement with the results of simpler tests based on LPS profiling of phage-resistant spontaneous mutants.

## Introduction

Exopolysaccharides (EPS) and capsules are generally considered to be the outmost surface structure of a bacterial cell. Many bacterial strains, however, do not produce any EPS or capsules. In Gram-negative bacteria lacking EPS, the outermost surface layer will consist of an external moiety of outer membrane (OM) lipopolysaccharides (LPS)^1^. LPS molecules are comprised of lipid A, core oligosaccharide and O-antigen (or O-polysaccharide; OPS). These structures play a role in the interactions of bacteria with various surfaces and molecules, including other bacterial or eukaryotic cells^2^, immunity factors^3–5^, enzymes^6^, and bacteriophages^7^. Bacterial surface oligo- and polysaccharides also contribute to structural stability of the outer membrane that may be relevant to bacteriophage penetration^8–11^.

The modulation of bacteriophage infectivity by OPS was first reported a long time ago^12^. Nevertheless, the importance of this host structure is still underestimated, however. In part, this is due to the fact that the bulk of data derived from “classical” bacteriophage biology was obtained from studies of model coliphages such as T-series (T1 to T7), as well as phage lambda and a few other phages that were propagated on laboratory rough, i.e., depleted in O-antigen biosynthesis, *E. coli* strains.

In some cases, the recognition of the receptors located on the intimate OM surface by phages can only be slightly affected by OPS production. For example, T5 phage can infect host cells *via* direct interaction of the phage receptor recognition protein (RBP) with the terminal (or secondary) receptor – OM transporter protein FhuA. Phage lateral tail fibers (LTF) interact with polymannose O-antigen of *E. coli* F, increasing T5 phage affinity to its host^13^. Nevertheless, an LTF-depleted phage T5 mutant was able to infect *E. coli* F with the same efficiency of plating (EOP) as was observed on the rough isogenic strain.

A growing body of evidence indicates that the majority of *E. coli* O-antigen types serve as a ‘perfect’ shield, completely or almost completely protecting the intimate OM surface from direct interaction with phage RBPs^14^. In turn, phages can penetrate this barrier using several different strategies^14,15^. Some phages interact with host LPS using enzymatically active RBPs^16^ to degrade the polysaccharide. Alternatively, phages may carry RBPs that recognize the OPS without degrading them. This latter strategy is employed by some T5-like bacteriophages^17^ and supposedly by many other siphoviruses^18^.

An important feature of bacteriophage infection strategies is to which extent interaction with OPS is essential for initiation of the downstream events of infection. Some phages are able to directly interact with their terminal receptors if these structures are exposed to them (e.g. on rough host strains that do not produce OPS). Examples of such phages are T5-like bacteriophages, phage lambda, and coliphage N4, plus many additional bacteriophages (see^14^ and references therein).

Some other bacteriophages, most often the podoviruses, employ a different strategy in which the OPS recognition is functionally connected with eventual DNA release from the virion^19^. So, the virus becomes strictly dependent on the presence of the OPS of particular type(s) on the cell surface. The interaction with OPS apparently generates a conformational signal that activates the viral adsorption device to enable it to interact with the terminal receptor leading to DNA release. Such phages often carry enzymatically active RBPs that degrade OPS or, less frequently, deacetylate it without destroying the polysaccharide backbone^20^.

Here we demonstrate that the infectivity of different bacteriophage against a panel of *E. coli* strain 4 s mutants deficient for O-antigen lateral modifications, OPS biosynthesis and core OS biosynthesis falls into distinct patterns compatible with the strategies described above. Using a modified high-throughput procedure for LPS analysis we also show that the effect of bacteriophage mediated selection on the ability of resistant host strains to produce O-antigen also depends on the strategy used by the virus.

## Results

### Development of a rapid LPS visualization protocol

Most protocols used for LPS visualization are relatively labor-intensive, slow and frequently lead to poor quality of the gels obtained. One of the main problems is the high sensitivity of conventional silver-staining protocols to any contamination of the LPS samples by substances containing amine groups, such as proteins. To tackle this problem we modified a procedure described by Tsai *et al*.^21^ that uses periodate-induced oxidation of saccharide moieties in LPS with subsequent silver staining. Our modified procedure differs from the one described in the work by Tsai *et al*.^21^ in several important respects. First, Tsai *et al*.^21^ used separate gel fixation and oxidation steps that lengthened the assay time. We successfully combined these steps into a single operation. Second, the original method used periodic acid as the oxidizer. We replaced it with sodium periodate that is less hygroscopic and easier to handle. The acidity of the fixing-oxidizing solution favorable for LPS oxidation is ensured by the acetic acid added. Application of the oxidation step resulted in a highly selective staining. This allowed us to use a very simple and rapid protocol for LPS extraction (see Material and methods section), with only partial protein removal by proteinase K treatment. The bacterial proteins remaining after Proteinase K digestion were unstained or poorly stained and did not interfere with the LPS analysis. The overall time required for the whole procedure, starting from a bacterial colony, is about 4 hours, with the number of the samples treated in parallel is mainly limited by the number of available wells on the acrylamide gel. These features allow us to use the protocol as a screening tool for many bacterial strains in parallel.

In order to test the performance of the modified protocol, we compared the LPS profiles of *E. coli* 4 s wild type (wt) and five mutants which are deficient for different genes involved in O-unit or core-OS biosynthesis. To do so, we used two previously described mutants 4 sI (*wclK*^*−*^) that lacks O-unit O-acetylation, and 4 sR (*wclH*^*−*^) completely deprived of O-antigen synthesis^22^. We also generated the mutants of *E. coli* 4 s strain deficient for lateral O-unit glycosylation (*gtr*^−^; 4 sGTR), outer core-OS synthesis (*waa*G^*−*^; 4 sG) and inner core-OS synthesis (*waa*C^−^; 4 sC).

**Figure 1:**
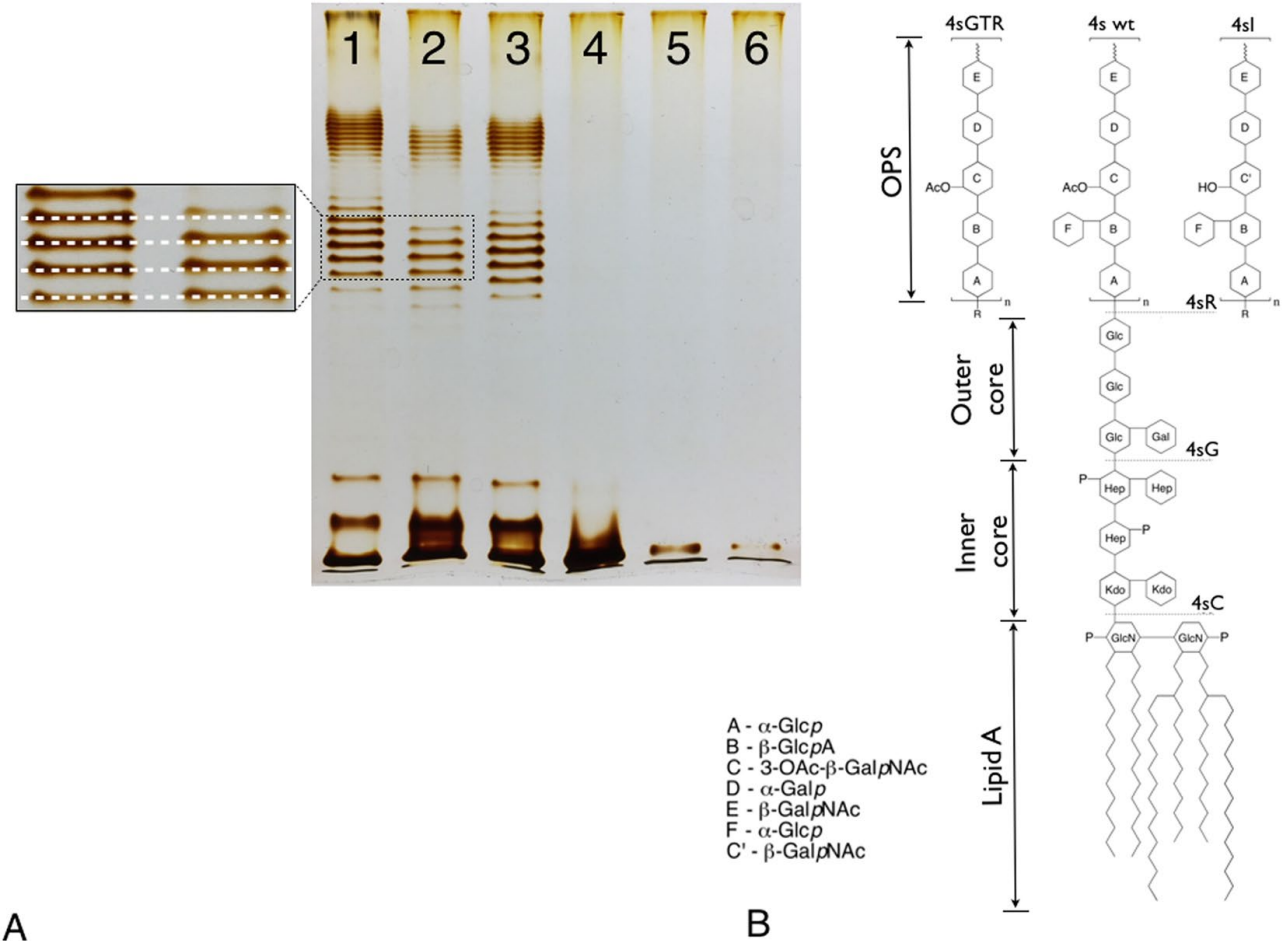
(**A**) LPS-profiles of *E. coli* 4 s and its mutants. Lines 1 – wt, 2–4 sI, 3–4 sGTR, 4–4 sR, 5–4 sG, 6–4 sC. The only band present in the strains 4 sR, 4 sG and 4 sC (lines 4–6) corresponds to the lipid A moiety with or without core OS; the ladders observed in the O-antigen producing strains (lines 1–3) correspond to populations of LPS molecules that differ by the length of their OPS chain by one O-unit. Note the non-even distribution of the OPS lengths that is due to specific control of this polysaccharide polymerization by Wzz proteins (see ref.^32^). An insert shows slight though visible band shift in 4 sI profile in respect to 4 s wt that occurs due to the absence of the O-acetyl group. Note much more pronounced bands shift in non-glycosylated LPS of 4 sGTR strain. (**B**) A schematic representation of *E. coli* 4 s LPS structure and its modification in the mutants.

In agreement with expected phenotypes, the strains *E. coli* 4 s wt, 4 sGTR (*gtr*^−^), and 4 sI (*wclK*^*−*^) produced similar LPS patterns (Fig. 1). A marked shift of the LPS bands can be observed in 4 sGTR (*gtr*^−^) LPS that is lacking lateral O-unit glycosylation and even in 4 sI (*wclK*^*−*^) strain OPS which lack only one small O-acetyl group rep 6 residues of repetitive O-unit; the band shift can be seen as well as it was observed previously with labor-intensive hot water-phenol-extraction of LPS by Westphal that takes about 2 days to perform^20,22^. Interestingly, OPS production seems to be reduced in 4 sI relative to 4 s wt.

At the same time, strains 4 sR (*wclH*^*−*^), 4 sG (*waaG*^*−*^) and 4 sC (*waaC*^−^) produced no OPS. However, the gel mobility of the LPS was different in agreement with expected full core-OS, inner core only or no core-OS, respectively (Fig. 1). Therefore, we concluded that the observed phenotypes of the *E. coli* 4 s mutants were in agreement with what we expected from their genotypes. Therefore, we felt that we could use these mutants to reveal the adsorption strategies of different bacteriophages.

### Analysis of bacteriophage susceptibility of the panel of *E. coli* 4 s mutants

We tested a series of bacteriophages (n = 40) from our laboratory collection for their ability to replicate on the strains mentioned in the previous section. Among these phages,.

15 were found to form plaques on at least one of the strains tested (Table 1). Only phage DT57C was able to infect all six strains. As it was demonstrated previously, this ability is due to the presence of lateral tail fibers (LTF) that recognize O-antigen of *E. coli* 4 s strain^17^. Other T5 related phages such as DT571/2, BF23 or Gostya9 that are genetically very close to DT57C^23^ were not able to infect OPS producing strain except for 4 sI, but infected all the rough strains regardless of their core-OS status. Interestingly, Gostya9 could infect with low efficiency the strain 4 sGTR. This may indicate that OPS glycosylation may contribute to the protective efficiency of the O-antigen layer.

**Table 1:** Sensitivity of *E. coli* 4 s and its mutants to bacteriophages.

		Strain ID, genotype and phenotype						Ref. or accession number
		4 s	4 sGTR	4 sI	4 sR	4 sG	4 sC	
		wt	gtrA−, gtrB−, gtrO22−	wclK^−^	wclH−	waaG−	waaC−	
Phage	Family/genus /note	O-acetylated and glycosylated OPS	Non-glycosylated but O-acetylated OPS	Non-acetylated, but glycosylated OPS	No OPS synthesis	No OPS, no outer core	No OPS, no core (Kdo only)	
DT57C	S/*T5virus*	+	+	+	+	+	+	NC_027356.1^17,23^
DT571/2	S/*T5virus*	−	−	+	+	+	+	KM979355.1^17,23^
BF23	S/*T5virus*	−	−	+	+	+	+	AAZ03642.1^46,47^
Gostya9	S/*T5virus*	−	Inh.	+	+	+	+	MH203051.1 Golomidova *et al*., 2018 – in press)
phiKT	P/NC/distantly related to T7	+	+	+	−	−	−	NC_019520.1^48^
G7C	P/*G7Cvirus*/N4-related	+	+	−	−	−	−	HQ259105.1^25^
Alt63	P/*G7Cvirus*/N4-related	+	+	+	−	−	−	^25^
Hf4s	P/NC/P22-related	+	+	Inh	−	−	−	NP
1.2/Zh	M/ND	−	−	+	+	+	+	NP
3.104/Zh9	P/NC/phiEco32-related	−	−	+	+	+	+	NP
4c	P/NC/phiEco32-related	−	−	+	+	−	−	NP
PEcoF	P/NC/phiEco32-related	+	+	Inh	+/−	−	−	NP
LAMP	P/NC/phiEco32-related	+	+	+	−	−	−	MG673519.1
9G	S/*Nonugvirus*	−	−	+	+	+	+	NC_024146.1^26^
St11Ph5	P/NC/G7C-like	−	−	−	Inh	Inh	inh	MG208881.1^24^

Phage family’s codes: S – Siphoviridae, M – Myoviridae, P-Podoviridae. NC – non-classified by ICTV at the moment of writing. ND – no data available. “+” – sensitive, efficiency of plating (EOP) >10^−2^ ;+/− EOP < 10^−4^; Inh – no plaques observed, but a turbid zone of inhibition of the lawn growth can be seen under the spot of concentrated lysate; “−” – resistant. NP – the phage characterization was not yet published.

Almost all the podoviruses tested demonstrated a dependence on OPS recognition for phage infection. Three phages (G7C, Hf4s, phiEcoF) recognize the O-acetylation, while PhiKT, LAMP and Alt63 need any OPS to be present on the cell surface, regardless of its acetylation status.

Interestingly, phiEco32-like phage 4c in contrast to other phages of this group, can infect the cells *via* direct recognition of the receptor on the intimate OM surface, most probably the outer core. Another surprising fact was the ability of St11Ph5 phage to infect, although with low efficiency, the rough *E. coli* 4 s mutants. This phage was initially isolated on the host producing O5-type OPS^24^. Since St11Ph5 is a closely related to G7C-phage, we expected that it should have similar infection strategy as phages G7C and Alt63 phages that are both strictly dependent on initial OPS recognition by their enzymatically active RBPs^20,25^ (and our unpublished observations). Counterintuitively, St11Ph5 phage proved to be able to bypass O-antigen recognition that is suggestive for the ability of this virus to recognize directly its secondary receptor.

### Detection of the LPS alterations selected by bacteriophage infection pressure

We suggest that the infection strategy used by а bacteriophage may determine the features of resistant host cell clones selected under the pressure of this phage, for example in course of phage therapy. In particular, the status of LPS production may be altered or remain intact. In that turn it may have a strong impact on the virulence of the phage-resistant mutant. In agreement with this assumption, G7C-resistant mutants described earlier^22^ were all deficient in OPS synthesis or O-acetylation. To further test this hypothesis, we obtained a series of the *E. coli* 4 s mutant clones selected for resistance to bacteriophages. For this comparison we have chosen three bacteriophages that are able to infect the wt strain but which feature different patterns of infectivity on the tested set of the mutant strains. These were phages DT57C, phiKT and LAMP. In addition to this series we obtained mutants of the *E. coli* up11 strain that was used for propagation of St11Ph5 phage. This was performed to test the hypothesis mentioned above that in contrast to closely related G7C-like bacteriophages, St11Ph5 phage may infect bacterial cells independently of O-antigen recognition. This should yield different patterns of LPS alterations in the host mutants selected for resistance to this virus compared to the features of G7C-resistant mutants^22^.

For each bacteriophage we analyzed 5–6 mutant host clones. These clones were purified by repeated single colony isolation and repeatedly checked for their resistance to the corresponding phage. We also checked for absence of emerging phages able to grow on the original host strain to avoid pseudolysogenic or carrier-state cultures^26,27^. All the cultures obtained were then analyzed by rapid LPS SDS-PAGE profiling.

**Figure 2:**
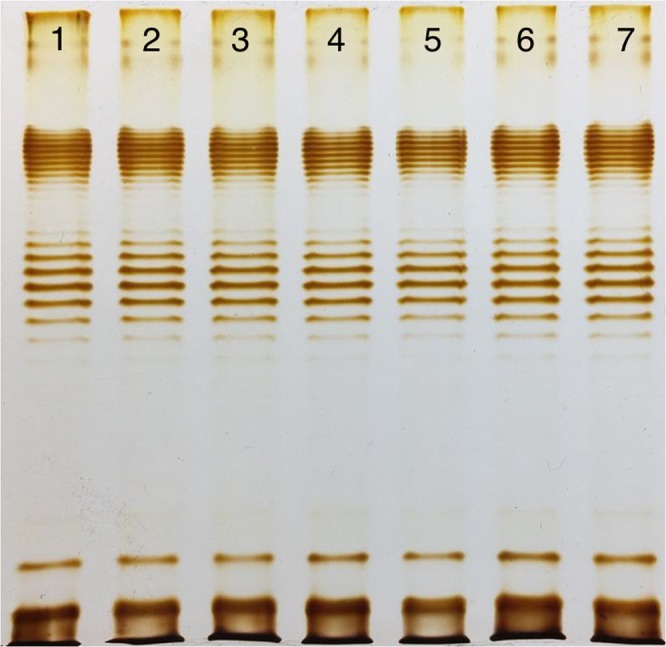
LPS profiles of *E. coli* 4 s (line 1) and its mutants, selected for resistance to the phage DT57C (lines 2–7). The LPS profiles of the mutant clones are identical to the wt strain that is compatible with the infection strategy of this T5-like phage, able to infect the cells *via* the direct recognition of its secondary proteinaceous receptor (BtuB).

The LPS profiles of all the phage resistant clones selected by DT57C phage were indistinguishable from the ones of the wild type (Fig. 2). It matched our expectations based on the data on the adsorption strategy used by this phage^17^. We suggested that in this case the resistance was due to the modification of the phage secondary receptor BtuB protein. We sequenced *btuB* gene in 2 of our mutants and found that in both cases mutations that potentially inactivate the gene (data not shown). We complemented mutant inactivated *btuB* with an wt allele from the plasmid and this yielded to partially restored phage sensitivity. The EOP observed on the complemented strains was about 10^−3^ compared to the wt strain, and the plaques were small and turbid (Fig. S1). However, no alterations of the bacterial lawn of the non-complemented strains was observed even as found under the concentrated phage stock spots (Fig. S1).

**Figure 3:**
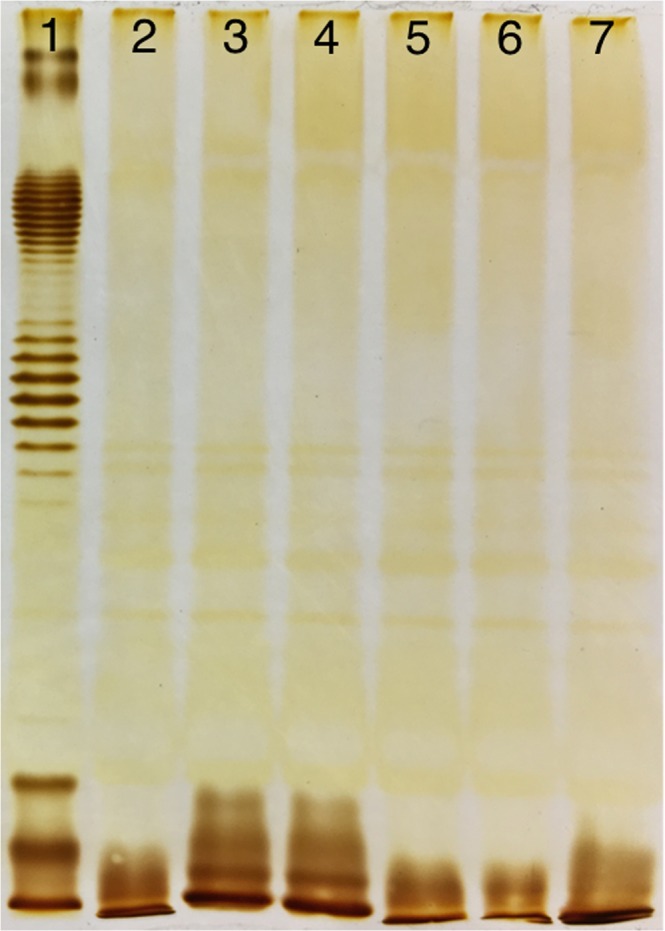
LPS profiles of *E. coli* 4 s (line 1) and its mutants, selected for resistance to the phage phiKT (lines 2–7). The resistant clones are depleted of O-antigen synthesis that is in good agreement with the infection strategy of the phage, which strictly depends on O-antigen recognition for enabling the interaction with the unknown secondary receptor, triggering DNA ejection.

**Figure 4:**
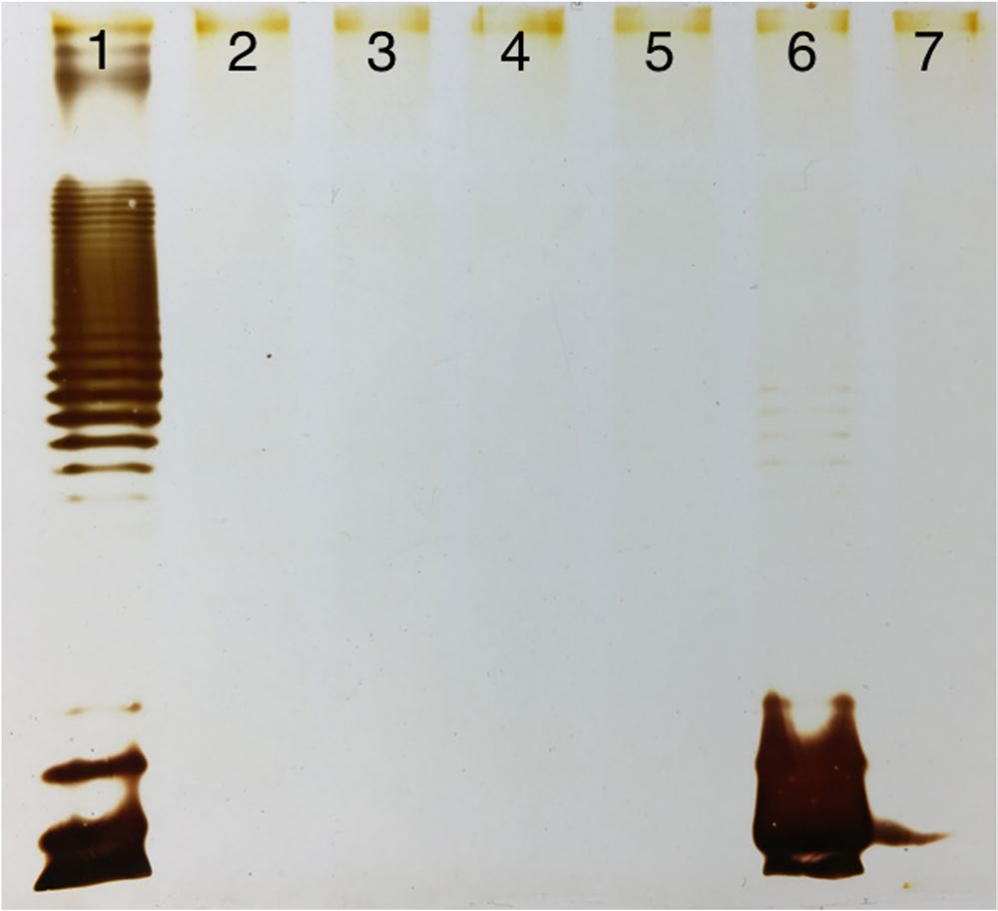
LPS profiles of *E. coli* 4 s (line 1) and its mutants, selected for resistance to the phage LAMP (lines 2–7). Note that one of the mutants (line 6) produces a lot of single LPS molecules with a O-unit with very low amount of longer OPS molecules. Phage LAMP, just as phiKT phage, depends on the initial O-antigen recognition for all subsequent events of the host cell infection.

In contrast to DT57C-resistant clones, the mutants selected for phiKT resistance were completely depleted of O-antigen biosynthesis (Fig. 3). The same pattern was observed in all but one of the LAMP-resistant mutants. One of the clones produced large quantities of LPS with a single O-unit, with very small amount of longer chain molecules (Fig. 4). In both cases, the observed LPS patterns were in good agreement with phage activity pattern against the LPS mutants set.

**Figure 5:**
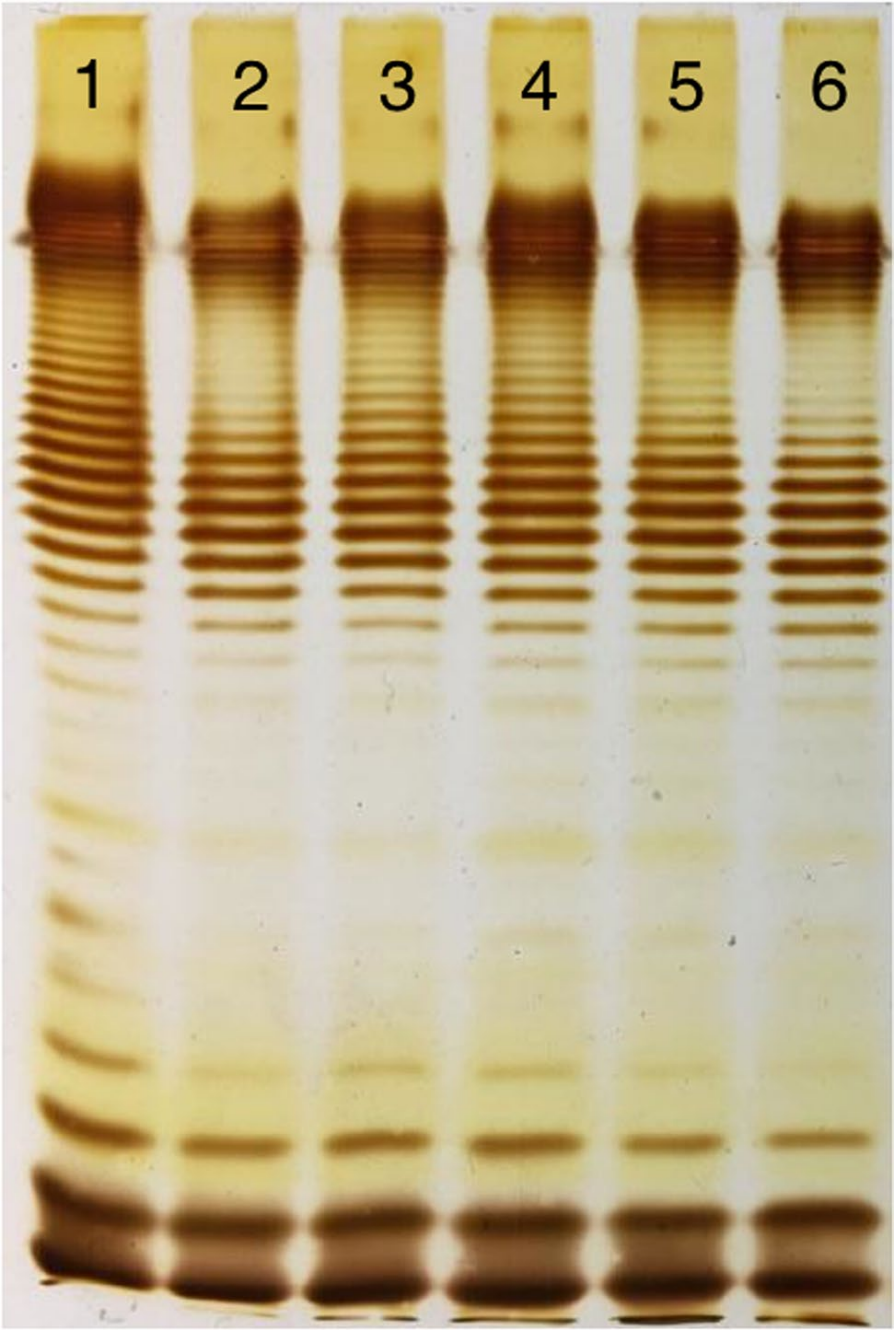
LPS profiles of *E. coli* UP11 (line 1) and its mutants, selected for resistance to the phage St11Ph5 (lines 2–7). The lack of O-antigen alterations in the mutant clones is suggestive of the phage ability to infect the host cell bypassing the OPS recognition.

Finally, the mutant clones of *E. coli* UP11 host strain selected for resistance to St11Ph5 phage were all identical to the corresponding wt strain by their LPS profiles (Fig. 5). This result is suggestive for the O-antigen independent recognition of the receptor by this phage both on UP11 cells and on 4 s cells.

## Discussion

Judging by the recent literature^28–30^, the polysaccharide silver staining using periodate oxidation step first proposed by Tsai *et al*.^21^ is now nearly forgotten, and many researchers use some commercial kits dedicated for protein staining to try and stain LPS. Frequently the combination of such a staining with simplified LPS extraction protocols, such as a simple proteinase K treatment, lead to poor quality gels that are not suitable for detection of slight modifications of LPS patterns that may reflect O-unit structure alterations (e.g. enabling or disabling of the lateral modifications). The classical LPS extraction methods such as the Westphal procedure^31^, that results in high purity LPS preparations suitable for production of high resolution gels (see for example Knirel *et al*.^22^), but they are labor intensive and time consuming. The protocol described here, optimized for high throughput, allows production of high quality LPS gels roughly 15 times faster than with the conventional Westphal procedure. At the same time, our modified LPS visualization procedure is apt not only to reveal major alterations in LPS profiles but also to detect even subtle variations in repetitive OPS unit (O-unit) structure such as lack of O-acetylation. We also confirmed that the phenotype of the mutants 4 sG and 4 sC correspond to the expected effect of modifications introduced in their genomes. The OPS alterations of 4 sGTR and 4 sI were previously checked using NMR spectroscopy^22^.

We also noticed that the OPS production in *E. coli* 4 sI strain was lower than in 4 s or 4 sGTR. This effect was repeatedly observed in multiple experiments. Since our procedure does not include any steps of LPS precipitation or other physical separation of the cell lysate fractions, we conclude that disabling of the O-unit O-acetylation indeed results in the reduced O-antigen biosynthesis. This is not surprising since O-unit O-acetylation precedes the O-antigen polymerization by Wzy polymerase^32^. The modification of the polymerase substrate therefore may reduce the efficacy of the reaction. In contrast to O-acetylation, the glycosylation is introduced after the polymerization and thus does not influence the level of O-polysaccharide synthesis. This observation highlights the performance of our LPS profiling procedure to detect alterations that can be relevant to phage sensitivity of the cells.

To test how LPS profiling of phage-resistant mutants may be predictive for the bacteriophage adsorption strategies, we evaluated the effect of several O-antigen alterations programmed in well-defined *E. coli* 4 s mutants on the activity of multiple bacteriophages (Table 1). Among the phages that are able to infect the wt *E. coli* 4 s strain, we observed two possible strategies: (1) phages that require initial O-antigen recognition for the infection (e.g. G7C, PEcoL, phiKT), and (2) phages that are potentially able to interact with their receptors on the cell surface directly, but require specific adaptations for penetration through the O-antigen shield (DT57C). For bacteriophages G7C and DT57C, these strategies were confirmed by more sophisticated experimental analysis^17,20^.

We therefore expected that the phages with strategy 1 will select for the resistant host clones with the phage secondary receptors altered of absent, but the O-antigens of such clones are expected to remain intact. At the same time strategy 2 would lead to an expectation that phage resistant mutants will be frequently depleted of O-antigen synthesis (rough strains) or have an altered O-unit structure. The results of experimental LPS profiling of the host mutants selected for the resistance for phages DT57C, phiKT and PEcoL were in complete agreement with these expectations.

In the case of DT57C-resistant mutants, no LPS modification was observed. At the same time the inactivation of the phage secondary receptor was confirmed by both *btu*B gene sequencing and complementation. Although full complementation was not achieved, and the complemented host poorly supported the growth of the phage, the resistance of the culture containing an empty vector was complete. We speculate that the observed decrease in EOP of the phage on the complemented strains could be due to too greatly elevated synthesis of BtuB precursor protein from the high copy number plasmid, which may cause biochemical effects that hinder its incorporation into the outer membrane and/or to the production of a high number of the molecules not connected to TonB energy transducing protein that is involved in infection process of many T5-related phages^33,34^.

Interestingly, one of the LAMP-resistant clones (Fig. 3, line 6) produced LPS with only one O-unit instead of longer O-polysaccharide chain. Only a small fraction of longer LPS molecules was synthetized. These observations suggest that this podovirus requires interaction with polymeric O-antigen for triggering the subsequent events of infection.

The ability of the bacteriophage St11ph5 to infect rough mutants of *E. coli* 4 s, although with low efficiency, motivated us to analyze the interactions of this virus with its original isolation host, *E. coli* UP11. This bacterial strain was isolated from a patient with urological infection and it proved to be particularly difficult to find the phages against it^24^. We were able to isolate only two viruses infecting this strain, one of which was found to be closely related to G7C phage^24^ that was previously isolated from horse feces^25^. The adsorption strategy of G7C phage has been characterized in considerable detail^20^. This virus interacts with *E. coli* 4 s O-antigen by its enzymatically active tail spike protein gp63.1 that removes lateral O-acetyl groups leaving the O-polysaccharide backbone intact. Nevertheless, this reaction is essential for G7C phage infectivity. Gp63.1 protein is connected to the virion neck *via* gp66, a fiber protein. Therefore gp63.1 and gp66 form a branched receptor recognition structure. The results by Prokhorov *et al*.^20^ and our unpublished data strongly suggest that the conformational signal, emerging as the result of gp63.1 interaction with its substrate (O-acetylated O-polysaccharide), is transmitted to the phage neck *via* gp66 and is necessary for activation of the particle for DNA delivery into the cell.

The comparative analysis of G7C and St11Ph5 genomes indicate that the latter phage also has a branched adhesin composed of the structurally similar proteins^24^. Although the predicted activity of St11Ph5 phage gp78 (an analog of gp63.1) was lyase, and not esterase (deacetylase), in some G7C-like phages gp63.1 is replaced by an alternative allelic variant with predicted lyase activity^25^ (and our unpublished data). We expected to find in St11Ph5 essentially the same infection strategy as in G7C that yield altered LPS structure in the resistant host clones^22^. To our great surprise, this was not the case, and all the resistant clones had an identical LPS profile to the wt strain. The mobility of the bands was exactly the same, indicating that not only the level of O-antigen synthesis and its length pattern were conserved but also O-unit structure remains unaltered. We should therefore conclude that the infection strategy of St11Ph5 is different from that of G7C despite the similarity of the adsorption apparatus organization in these viruses. Bacteriophage N4 is also able to infect rough *E. coli* strains *via* direct recognition of NfrA protein by its tail sheath protein gp65^35^, the homolog of N4 gp65 was also found in phiAxp-3 *Achromobacter* phage where it also acts as a receptor-recognition protein binding to the host LPS^36^. G7C phage and numerous related viruses, including St11Ph5, do not have any gp65 homolog. Their adsorption locus located between conserved genes related to N4 phage genes 63 and 67, is comprised by the tail spike (e.g. gp63.1) and tail fiber (gp66) protein genes^20,24,25^. These marked differences were believed to be connected with a different cell recognition strategy in which the interaction with and enzymatic modification of O-polysaccharide is an essential step of the phage infection. The data obtained here contradict this hypothesis. We can conclude that in N4-related phages the strategy of host cell recognition is a species-specific or even a strain-specific trait depending on quite subtitle modification of the adsorption device rather reflecting its overall architecture. An in-depth comparative study of G7C and St11Ph5 infection process may help to reveal the key mechanism of the virion activation.

The LPS structure of the emerging phage-resistant clones may also have an implication in bacterial virulence alterations during the phage therapy^37^. Although the resistance to bacteriophages dependent on O-antigen recognition may emerge with high frequency (e.g. 10^−4^ – 10^−3^ of G7C resistant clones in the cultures of *E. coli* 4 s^22^), these bacteria lose an important protection against serum and other immunity factors^38,39^ and therefore can be expected to have decreased virulence. If the pathogen population is temporally protected from the immune system (e.g. in an abscess or within a biofilm) but experiences the phage infection pressure due to phage therapy, the phage-resistant clones with impaired virulence may outcompete the wt cells and thus reduce the transfer of the infection to other sites.

To make a general conclusion, we highlight that screening of the LPS status of phage-resistant clones may be a simple and valuable test for characterizing new phage isolates and it may reveal their cell recognition strategy and will help to select the unusual strains for deeper examination. The results of this test may also be valuable for prediction of the therapeutic potential of new candidate phage strains although more data on correlation of phage efficacy in therapy and its infection strategy is required. The rapid and high throughput protocol for LPS visualization that we present here is easy to establish in almost any laboratory and we believe that it will facilitate the introduction of the phage resistant clones LPS profiling as a routine procedure of description of the novel phage isolates infecting Gram-negative bacteria.

## Methods

### Bacterial and bacteriophage strains

The *E. coli* 4 s strain was isolated from the horse feces in 2006^40^ and characterized previously^22^ (Knirel *et al*. 2015). The mutants of this strain, 4 sI and 4 sR, were also described previously^22^. Other mutants were obtained in the course of this work. The *E. coli* UP11 strain was isolated the clinical bacteriological laboratory in the Central Research Institute of Epidemiology, Federal Supervision Service for Consumer Rights Protection and People’s Welfare, Moscow, Russia, from urine sample of an urological patient in 2015. This strain found to belong to O5 serotype and LPS core type 1 (to be published elsewhere).

Bacteriophages were from the collection of our laboratory. Some of them were characterized including by whole genome sequencing. Others are Uncharacterized and/or unpublished. The references for publications and genomic sequences of bacteriophages are given in the Table 1.

All bacterial strains were cultured on LB medium. Bacterial and phage growth, phage titration and selection of phage resistant mutants were performed using standard techniques described in Golomidova *et al*.^17^.

### LPS extraction and electrophoresis

LPS was extracted using a Proteinase K micro digestion protocol adapted from Hitchcock P. J. (1984)^41^. Briefly, a bacterial colony grown on an LB plate was transferred to 1 ml of physiological saline. Cells were pelleted by centrifugation at 10000 g in a table-top microcentrifuge for 30 s, resuspended in 1 ml of physiological saline, pelleted again and resuspended in 50 ul of lysis buffer (2% w/v of SDS, 4% v/v of 2-mercaptoethanol, 10% v/v glycerol, 1 M Tris-HCl (pH 6.8) up to the desired volume and bromophenol blue to deep blue color). Resuspended cells were incubated at +95 °C for 10 min, cooled to room temperature, then 10 ul of 2.5 mg mL^−1^ Proteinase K solution made in the lysis buffer was added (Proteinase K 10 mg mL^−1^, stock solution can be made in 20% glycerol and kept at −20 °C; to prepare Proteinase K working solution the stock solution should be diluted 4-fold with the lysis buffer). This mixture was further incubated at +56 °C for 1 hour in a heating shaker. The preparation obtained was directly loaded on a conventional protein SDS polyacrylamide gel^42^ with 12% of acrylamide (19:1 acrylamide:bisacrylamide), loading 2–5 ul per well of the gel cast in a Protean III device with 10- or 15-teeth combs (Bio-Rad, USA). The gels were run in Bio-Rad Protean III cell at a constant 20 mA current in Tris-glycine-SDS buffer.

All the solutions were prepared from analytical grade reagents and were made from stocks right before use; all incubations and washes were done in plastic food containers, solutions removed by aspiration. For development of the LPS profile, the gel was removed from the electrophoresis cell and put in 25 ml of fixer-oxidizer solution (40% v/v ethanol, 5% v/v acetic acid, 1% w/v sodium periodate, MilliQ DI water up to 1 v). After 15 minutes of incubation in this solution on an orbital platform shaker, the gel was washed with distilled water thrice (7 minutes each wash step) to remove all remaining Tris that would otherwise give a high background staining. Then the stain solution (15 ml MilliQ DI water, 1.4 ml 0.1 M NaOH, 100 ul concentrated 35% w/w ammonia, 250 ul of 20% silver nitrate) was poured onto the gel. After a 10-minute incubation on an orbital shaker (75 rpm), the stain solution was removed and the gel was washed thrice with DI water (25 ml each time, 15 s of each washing). This step is found to be crucial for getting good signal-to-background ratio, extended washing removes too much silver and reduces the contrast and sensitivity. After all washes, a pre-warmed 40 °C developer solution was added to the gel (100 ul 3% citric acid, 25 ul 30% formaldehyde, 50 ml MilliQ DI water). The tray with the developing gel was incubated on a platform shaker avoiding extensive illumination (e.g. direct sunlight) until the band pattern is well developed; for full development the background between the lanes should become slightly stained. The reaction was stopped by aspiration of the developer and washing the gel in distilled water. The gels were digitally photographed under a layer of water in a transparent tray on a light table.

### Generation of *E. coli* 4 s gene knock-out mutants and selection of the phage resistant clones

All the mutants were generated by a recombineering procedure^43^. The pKD46 plasmid was used as a source of lambda-Red proteins, pKD3 plasmid was used as a template for PCR generation of mutagenizing DNA fragments. The recombinants were selected for kanamycin resistance and verified by the targeted sequencing of the corresponding locus. No step for deletion of the inserted Km cassette was applied.

For deletion of the *gtr* locus comprised by three genes *gtrA, gtrB, gtrO22* that encode for enzymes required for lateral glycosylation of the O22-type O-unit^22^ we used primers gtrO22.FP4 (TATACAGTATCGTCAAACATGAGAGTCGATCAAGTTattccggggatccgtcgacc) and grtO22.RP1 (AAATTAGCCTGGCTATGCCAGGCTTCCACTCACTTAgtgtaggctggagctgcttc) where capital letters indicate sequences matching the target and line letters stand for the nucleotides of the sequences hybridizing to the plasmid PCR template.

Gene *waaG* is responsible for addition of the first hexose residue of the outer core in *E. coli*^44,45^. For knockout of this gene we used the primers waaG.FP1 (TAAATTACTGCCCTCCTCCACGACAGGTACGTCGTTATGAgtgtaggctggagctgcttc) and waaG.RP4 (GATATCCGCCGCTTTCTCTGGCAGACTGTATAAATCTTGTattccggggatccgtcgacc). This deletion was performed in 4 sR (wcaH-) strain because the transformation efficiency in this strain is much higher than in O-antigen producing wild type (wt) strain. Therefore, not all the loci could be successfully modified in the wt background. At the same time the resulting modification of the cell surface (lack of both outer core and O-antigen) is independent of the ability of the cell to make O-unit blocks.

Gene *waaC* is responsible for addition of the first hexose of the inner core^44,45^. For its knockout we used the primers waaC.FP1 (GGAAGAACTCAACGCGCTATTGTTACAAGAGGAAGCCTGAgtgtaggctggagctgcttc) and waaC.RP4 (AACTTATTGGTTATTATTTCAAGATTTAAATTTTGAAAAAattccggggatccgtcgacc). As for *waaG* deletion, the strain 4 sR was used.

The selection of the host strain spontaneous mutant clones was performed as it was described previously^17^. Briefly: the phage agar plates were prepared as conventional double-layer plates (e.g. as used for phage titration). However, instead of bacterial culture ca. 10^8^ PFU per plate of an appropriate filter-sterilized phage stock was added to the top agar. After the top agar solidification, 100 ul aliquot of a mid-log phase culture (or its appropriate dilution, if the frequency of the resistant clones is high) was gently spread over the agar using a glass spreader. The plates were incubated overnight, resistant clones were picked with toothpick and isolated by subcloning. Important: for subcloning the colonies should be resuspended in liquid medium, vigorously vortexed and appropriate dilutions should be plated with spreader. The streaking with bacterial loop is frequently not sufficient for isolation. The purified resistant clone cultures were checked for the resistant phenotype and for absence of emerging phages able to form plaques on the lawn of the original host.

### Analysis and complementation of BtuB mutations

For amplification and sequencing of *btuB* gene we used primers BtuB-F (CCAACGTCGCATCTGGTTC), BtuB-R (GATCTCGTCATAGACCGA). In addition, primer BtuB-F2 was used to sequence the central part of the PCR fragment. To create the plasmid pBtuB, the PCR fragment obtained from the primers BtuB-F and BtuB-R was cloned into pGEM-T vector (Promega, USA), according to the manufacturer’s instructions. The insert orientation under the control of lac-promoter was selected. The plasmid was checked by the Sanger sequencing of the insert. Complementation was performed as it was described earlier^22^.

## Supplementary information

Supplementary figure S1
