# Supplementary material for: High-throughput LPS profiling as a tool for revealing of bacteriophage infection strategies
Source: Sci Rep. 2019 Feb 27;9:2958. doi: 10.1038/s41598-019-39590-8 (PMC6393563; doi:10.1038/s41598-019-39590-8)
Supplement: Supplementary file 1 — Supplementary figure S1 [file 41598_2019_39590_MOESM1_ESM.pdf]

High-throughput LPS profiling as a tool for revealing of bacteriophage infection strategies

Eugene E. Kulikov[1,2], **Alla K. Golomidova**[1], **Nikolai S. Prokhorov**[1,•], **Pavel A. Ivanov**[1], **Andrey V. Letarov**[1,2,3,*]
[1]Winogradsky Institute of Microbiology, Research Center of Biotechnology of the Russian Academy of Sciences, prosp. 60-letiya Oktyabrya, 7/2, 117312, Moscow, Russian Federation

[2]Moscow Institute of Physics and Technology, Institutskiy per., 9, Dolgoprudny, Moscow Region, 141701, Russian Federation

[3]Faculty of Biology, Lomonosov Moscow State University, ul. Leninskie Gory, 1, 119991, Moscow, Russia

• present address: Department of Biochemistry and Molecular Biology, Sealy Center for Structural Biology and Molecular Biophysics, University of Texas Medical Branch, 301 University Bulevard, Galveston, TX, USA

Suplementary information

Figure S1. Complementation of the *E. coli* 4s mutant clone selected for phage DT57C resistance by plasmid expressing BtuB protein. The plaques formed on the complemented strain are very turbid; therefore single plaques are hard to see on the photo (some of them indicated by the arrows), however these plaques are clearly visible on the original plate. The EOP on the complemented strain was reduced by 3 orders of magnitude compared to the wt strain. Note that no phage growth is detectable on the non-complemented mutant strain lawn even under the spot of the concentrated phage stock.

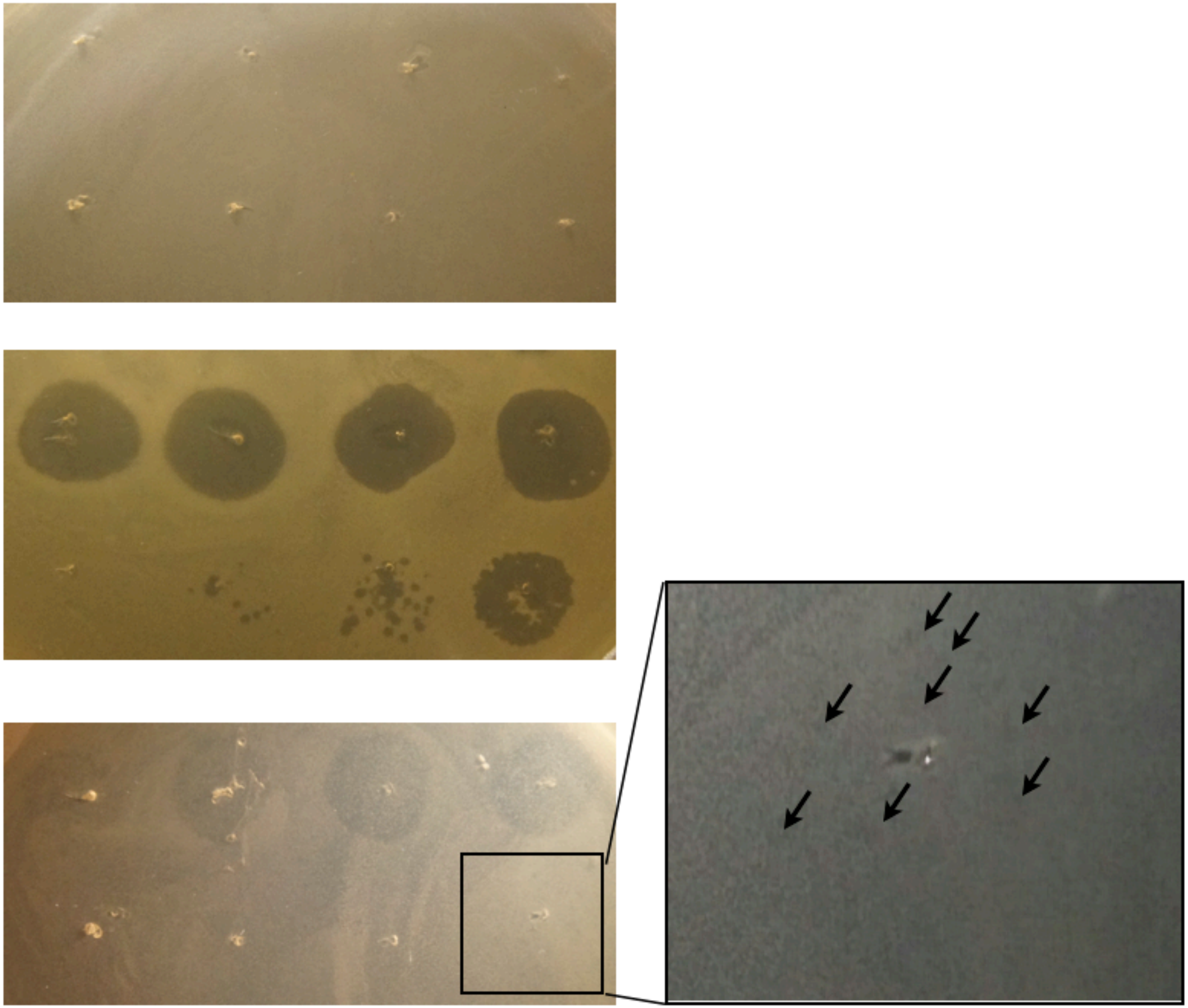

4s_DT57C-R

4s wt

4s_DT57C-R:
:pBtuB
